# Yellow Fever Molecular Diagnosis Using Urine Specimens during Acute and Convalescent Phases of the Disease

**DOI:** 10.1128/jcm.00254-22

**Published:** 2022-08-02

**Authors:** Izabela Maurício de Rezende, Gabriela Fernanda Garcia Oliveira, Thaís Alkifeles Costa, Aslam Khan, Leonardo Soares Pereira, Tayrine Araújo Santos, Pedro Augusto Alves, Carlos Eduardo Calzavara-Silva, Olindo Assis Martins-Filho, Andréa Teixeira-Carvalho, Angelle Desiree LaBeaud, Betânia Paiva Drumond

**Affiliations:** a Laboratory of Viruses, Microbiology Department, Biological Sciences Institute, Federal University of Minas Gerais, Belo Horizonte, Minas Gerais, Brazil; b Department of Pediatrics, Division of Infectious Disease, Stanford University School of Medicine, Stanford, California, USA; c Eduardo de Menezes Hospital, Belo Horizonte, Minas Gerais, Brazil; d Immunology of Viruses Diseases, René Rachou Institute, Oswaldo Cruz Foundation/FIOCRUZ MG, Belo Horizonte, Minas Gerais, Brazil; e Cellular and Molecular Immunology, René Rachou Institute, Oswaldo Cruz Foundation/FIOCRUZ, Belo Horizonte, Minas Gerais, Brazil; f Integrated Group of Biomarkers Research, René Rachou Institute, Oswaldo Cruz Foundation/FIOCRUZ, Belo Horizonte, Minas Gerais, Brazil; St. Jude Children's Research Hospital

**Keywords:** yellow fever virus, yellow fever, virus surveillance, diagnosis, urine

## Abstract

Prior studies have demonstrated prolonged presence of yellow fever virus (YFV) RNA in saliva and urine as an alternative to serum. To investigate the presence of YFV RNA in urine, we used RT-PCR for YFV screening in 60 urine samples collected from a large cohort of naturally infected yellow fever (YF) patients during acute and convalescent phases of YF infection from recent YF outbreaks in Brazil (2017 to 2018). Fifteen urine samples from acute phase infection (up to 15 days post-symptom onset) and four urine samples from convalescent phase infection (up to 69 days post-symptom onset), were YFV PCR-positive. We genotyped YFV detected in seven urine samples (five collected during the acute phase and two collected during the YF convalescent phase). Genotyping indicated the presence of YFV South American I genotype in these samples. To our knowledge, this is the first report of wild-type YFV RNA detection in the urine this far out from symptom onset (up to 69 DPS), including YFV RNA detection during the convalescent phase of YF infection. The detection of YFV RNA in urine is an indicative of YFV infection; however, the results of RT-PCR using urine as sample should be interpreted with care, since a negative result does not exclude the possibility of YFV infection. With a possible prolonged period of detection beyond the viremic phase, the use of urine samples coupled with serological tests, epidemiologic inquiry, and clinical assessment could provide a longer diagnostic window for laboratory YF diagnosis.

## INTRODUCTION

After decades of causing small outbreaks in remote areas of the globe, yellow fever virus (YFV) caused large outbreaks of yellow fever (YF) in African and South American countries from 2016 to 2019. In 2016, YFV spread through Angola and the Democratic Republic of Congo, resulting in 884 confirmed cases and 381 deaths (1). These cases were also imported to other countries, such as China (2). Later in 2016, the virus reemerged in Southern Brazil and caused 2,166 confirmed cases and 752 deaths from 2016 to 2019 (3–5).

The clinical course of YF is classically divided into three stages: (i) infection, characterized by viremia and occurrence of flulike symptoms; (ii) remission, when seroconversion is observed while fever and symptoms disappear; and (iii) intoxication, which is considered the severe form of YF, when symptoms reappear and patients experience hemorrhagic fever, multiorgan dysfunction, jaundice, oliguria, and anuria, among other symptoms (4, 6, 7). After these three stages, there is a convalescent phase of disease, which is characterized by prolonged weakness and fatigue lasting several weeks (7–11).

According to the World Health Organization (WHO), a YF suspected case is defined as any individual who presents with an acute febrile illness and jaundice development within 14 days after symptom onset. A YF probable case is defined by a suspected individual testing positive for YF IgM antibodies in the absence of YF vaccination or an epidemiological link with a YF confirmed case or outbreak (12). In both cases, laboratory testing must be performed to confirm YF diagnosis. YF laboratory testing is performed through molecular, virologic or serological methods using mainly serum collected in different phases of the infection, with preference for serum from the acute phase of infection (13). Serological tests targeting detection of anti-YFV IgM antibodies are recommended after seroconversion, often utilizing samples collected from the sixth day after the onset of symptoms onward. However, the detection of antibodies against YFV poses multiple challenges as serologic tests do not necessarily indicate an active YFV infection, cross-reactivity with other flaviviruses can occur, and routine serological tests for detection of IgM or IgG do not discriminate between immunologic responses created by natural infection or by the YF vaccine. The gold standard for laboratory diagnosis is real-time reverse transcriptase PCR (RT-qPCR) using whole blood or serum collected from days one to 10 post-symptom onset (DPS) (13). However, according to the WHO, there is a limited window of 10 DPS of YFV RNA detection in serum, and the use of alternative biological samples like urine may provide a useful approach for molecular diagnosis.

Prior studies have demonstrated prolonged presence of RNA in urine or saliva from other flaviviruses such as Zika virus (ZIKV) and dengue virus (DENV) (14, 15). Regarding the YFV, previous studies had demonstrated the presence of YFV RNA in urine samples when viral RNA is no longer detected in sera (16–18). One study demonstrated the presence of YFV RNA in urine up to 32 DPS in patients who recovered after YF disease and urine samples in some cases contained higher viral loads compared to blood samples collected on the same day (16). In another study, YFV RNA was detected in urine and semen samples from a naturally infected YF patient up to 21 DPS, when the serum was already RT-qPCR negative (18).

We evaluated the applicability of urine as a sample for YFV laboratory molecular detection by testing a range of samples collected from a large cohort of YF infected patients at acute and convalescent phases of YF infection during recent YF outbreaks in Brazil, from 2017 to 2018.

## MATERIALS AND METHODS

### Biological samples and ethics.

A total of 480 patients were attended at Eduardo de Menezes Hospital (HEM), Belo Horizonte, Brazil, a reference hospital for infectious diseases in Minas Gerais state, during the 2017 to 2018 YF outbreak. Routine YF laboratory diagnosis tests (PCR or ELISA) were performed at Reference Laboratory in Minas Gerais (Ezequiel Dias Foundation—FUNED). The YF laboratory diagnosis was confirmed through positive YFV PCR or detection of anti-YFV IgM, followed by negative anti-DENV and anti-ZIKV IgM tests. Of the 302 patients with confirmed YF, 60 patients provided consent and urine samples for the current study. Urine samples from the 60 patients enrolled in this study were obtained in different DPS (Table 1) and analyzed. One urine sample per patient was analyzed, and whenever available, serum samples were also analyzed (Table 1). Twenty-seven urine samples were collected during the YF acute phase (up to 15 DPS), and 33 urine samples were obtained during the convalescent phase (from 19 to 94 DPS) (Table 1). Urine and serum samples were collected at the hospital and kept in liquid nitrogen until adequate transport to Virus Laboratory/ICB/UFMG, where the samples were maintained in an ultra-freezer at −70°C until use. Out of 60 patients enrolled in the study, 58 patients had no record of YF vaccination, while two patients had been vaccinated against YF 10 days before symptom onset. This study was approved by the Ethics Committee for studies with human subjects on Human Research at René Rachou Institute/FIOCRUZ-MG on CAAE 65814417.0.0000.5091 and CAAE: 43000815.7.0000.5091.

**TABLE 1:** Urine and serum samples collected during the acute and convalescent phases of yellow fever infection tested for the presence of yellow fever virus RNA, using RT-qPCR^*a*^

ID	Symptom onset	YFV diagnosis (performed by the reference laboratory FUNED)^*b*^		RT-qPCR result (performed in research lab–Virus Lab/ICB/UFMG)			Vaccination date	YFV genotype
		DPS	PCR or ELISA	DPS	SERA	URINE		
1	3/3/18	10	PCR +	3	P	P	NV	NA
2	1/14/18	2	PCR +	4	P	P	NV	NA
3	1/22/18	3	PCR +	5	NA	P	NV	SA-I
4	1/28/18	3	PCR +	5	NA	P	NV	NA
5	2/12/18	4	PCR +	6	NA	P	NV	NA
6	2/1/18	8	PCR +	8	NA	P	NV	SA-I
7	2/26/18	3	PCR +	8	NA	P	NV	SA-I
8	2/12/18	3	PCR +	9	NA	P	NV	NA
9	1/6/18	6	PCR +	10	N	P	NV	SA-I
10	2/9/18	6	PCR +	13	N	P	NV	SA-I
11	1/18/18	6	PCR +	15	NA	P	NV	NA
12	1/16/18	10	PCR +	22	N	P	NV	NA
13	3/15/18	3	PCR +	28	N	P	NV	NA
14	1/22/17	5	PCR +	67	N	P	1/16/17	SA-I
15	1/15/17	3	PCR +	69	N	P	1/14/17	SA-I
16	1/20/18	5	PCR +	1	NA	N	NV	NA
17	2/4/18	NA^*c*^	PCR +	4	NA	N	NV	NA
18	1/22/18	2	PCR +	5	NA	N	NV	NA
19	2/15/18	NA^*c*^	PCR +	5	NA	N	NV	NA
20	1/28/18	33	PCR +	7	NA	N	NV	NA
21	2/23/18	1	PCR +	7	NA	N	NV	NA
22	3/16/18	NA^*c*^	PCR +	7	NA	N	NV	NA
23	2/5/18	3	PCR +	8	NA	N	NV	NA
24	3/5/18	NA^*c*^	PCR +	8	NA	N	NV	NA
25	1/12/18	NA^*c*^	PCR +	9	NA	N	NV	NA
26	1/22/18	5	PCR +	9	NA	N	NV	NA
27	1/12/18	10	PCR +	10	NA	N	1/16/18	NA
28	1/22/18	3	PCR +	11	NA	N	1/22/18	NA
29	1/28/18	9	PCR +	11	NA	N	NV	NA
30	1/21/18	10	PCR +	12	NA	N	1/24/18	NA
31	2/15/18	NA^*c*^	PCR +	15	NA	N	NV	NA
32	1/22/18	NA^*c*^	PCR +	19	NA	N	NV	NA
33	1/18/18	3	PCR +	21	NA	N	>20 DAYS	NA
34	2/5/18	6	PCR +	31	NA	N	NV	NA
35	1/16/18	4	PCR +	36	NA	N	NV	NA
36	1/25/17	2	PCR +	45	NA	N	NV	NA
37	2/26/18	NA^*c*^	PCR +	46	NA	N	NV	NA
38	1/22/17	NA^*c*^	PCR +	48	NA	N	1/12/17	NA
39	1/13/18	4	PCR +	48	NA	N	NV	NA
40	1/28/17	4	PCR +	50	NA	N	1/16/17	NA
41	1/15/17	NA^*c*^	PCR +	55	NA	N	NV	NA
42	1/22/17	3	PCR +	62	NA	N	1/10/17	NA
43	1/15/17	6	PCR +	64	NA	N	1/16/17	NA
44	1/17/17	5	PCR +	67	NA	N	1/16/17	NA
45	1/23/17	3	PCR +	68	NA	N	1/23/17	NA
46	1/22/17	5	PCR +	69	NA	N	1/22/17	NA
47	1/8/17	5	PCR +	69	NA	N	3/21/01	NA
48	1/8/17	24	ELISA +	69	NA	N	NV	NA
49	1/18/17	NA^*c*^	PCR +	73	NA	N	NV	NA
50	1/11/17	23	PCR +	73	NA	N	NV	NA
51	1/26/18	NA^*c*^	PCR +	77	NA	N	NV	NA
52	1/7/17	6	ELISA +	77	NA	N	NV	NA
53	1/7/17	1	PCR +	77	NA	N	NV	NA
54	1/10/17	5	PCR +	78	NA	N	1/11/17	NA
55	1/5/17	8	ELISA +	79	NA	N	1/14/17	NA
56	1/10/17	4	PCR +	81	NA	N	NV	NA
57	1/5/17	8	PCR +	81	NA	N	NV	NA
58	1/14/17	5	PCR +	84	NA	N	NV	NA
59	1/25/18	5	PCR +	85	NA	N	NV	NA
60	1/4/17	14	PCR +	94	NA	N	NV	NA

*^a^*The positive urine samples from yellow fever acute phase (until 15 DPS) were highlighted in gray. Positive urine samples from yellow fever convalescent phase (after 16 DPS) were highlighted in blue. ID: patient identification. DPS: days post-symptom onset. P: positive for the presence of yellow fever virus RNA by RT-qPCR (16). N: negative for the presence of yellow fever RNA (16). NA: not available. NV: not vaccinated (patients did not report a history of vaccination against yellow fever).

*^b^*YFV diagnosis confirmed by Central Reference Laboratory (FUNED) linked to Ministry of Health and Health Secretary of Minas Gerais state.

*^c^*Date of exam’s result is not available; however, the positive YF case was confirmed.

### YFV RNA screening.

A total of 140 μL of each urine sample was used for total RNA extraction, using the QIAmp Viral RNA minikit (Qiagen), following the manufacturer’s instructions. Total RNA (5 μL) was used in an RT-qPCR targeting part of the YFV 3’UTR region of the YFV genome (19), for YFV RNA screening. Positive samples were then used for YFV genotyping, as described below. Whenever possible, YFV RNA was also screened in serum from patients who had YFV RNA detected in urine.

### YFV genotyping.

Urine samples presenting YFV RNA were genotyped (20). For this, 5 μL of total RNA obtained from a positive urine sample was submitted to a different RT-qPCR, using primers targeting the NS5 region (21), and amplicons were used for nucleotide sequencing (22). The sequences generated were then aligned with a sequence panel previously used (20), using Clustal W, implemented on Mega7 (22). The Maximum-likelihood tree was generated using Kimura-2-parameters nucleotide substitution model with gamma distribution and 1,000 bootstraps replicates, using MEGA7 (23).

### Data availability.

Sequence data generated in this study were deposited in GenBank under accession numbers OM692343 to OM692349.

## RESULTS

Of 60 analyzed urine samples, 15 (25%) were positive for the presence of YFV RNA (Ct range: 29–33) (Table 1). Eleven urine samples were taken from the acute phase (11/27 = 40.7%) (collected from 2 to 15 DPS; Table 1) and four urine samples were taken from the convalescent phase (4/33 = 12.1%) (collected at 22-, 28-, 66-, and 69-DPS; Table 1). From eight patients we were able to analyze serum collected on the same day of the analyzed urine sample. In two patients (ID 1 and ID 2; Table 1) YFV RNA was detected in serum and urine samples collected on the same day (3 and 4 DPS, respectively; Table 1). Six patients (IDs 9, 10, 12, 13, 14, and 15; Table 1) tested positive in urine while YFV RNA was no longer detected in serum samples collected on the same day as urine (Table 1).

**FIG 1:**
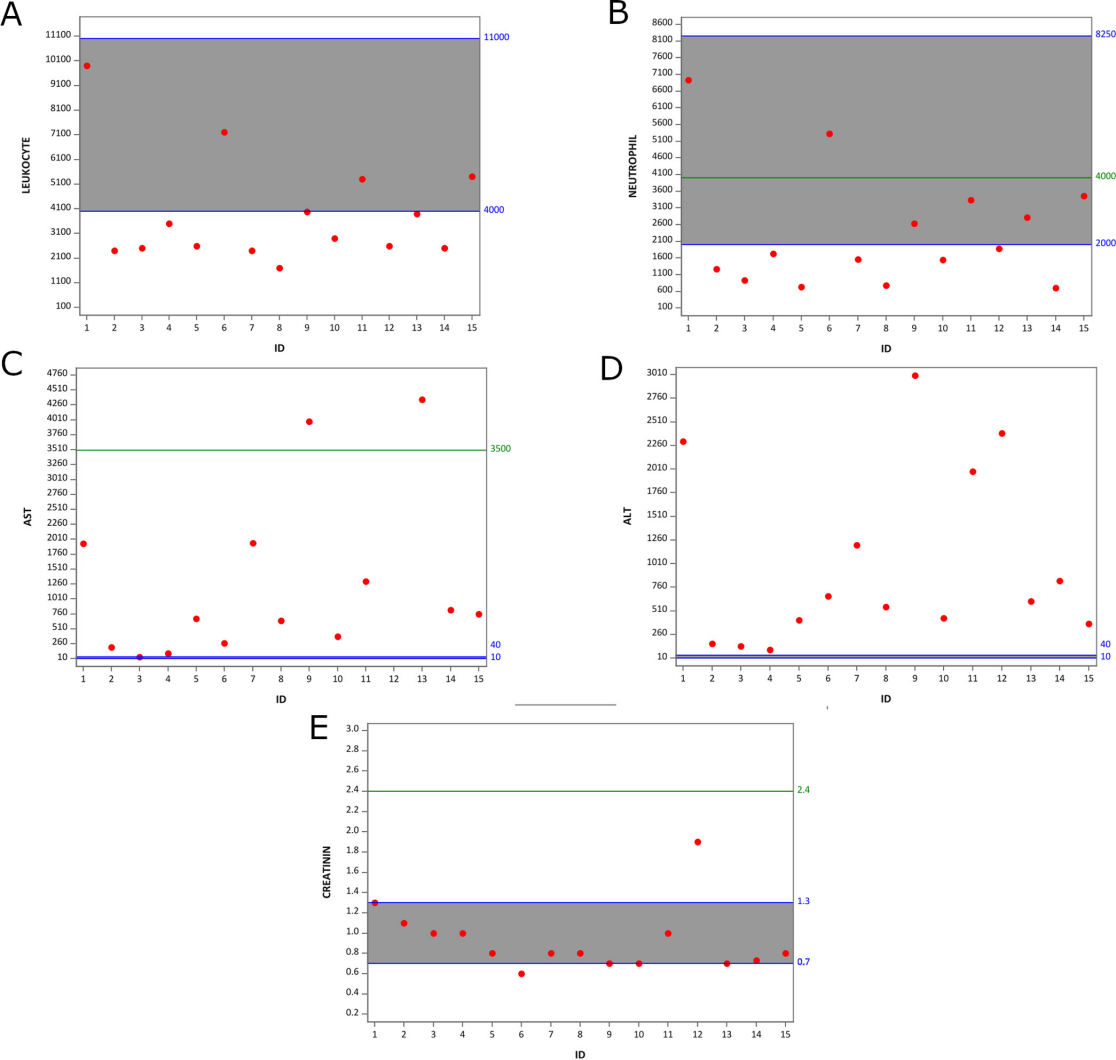
Plot of laboratory exams values of patients from whom YFV RNA were detected in urine samples. Shown here are leukocyte count (mm^3^) (A), neutrophil count (mm^3^) (B), ALT (UI/L) (C), AST (UI/liter) (D), and creatinine (mg/dL) (E). Normal range values are shown between blue lines and highlighted in gray. The cutoff described by Kallas et al. (2019) for developing severe YF disease is represented by the green line. Data from P2, P3, P4, P5, P7, P8, and P13 were collected 3 DPS. Data from P9 and P14 were collected 5 DPS. Data from P10 and P11 were collected 6 DPS. Data from P1, P6, and P15 were collected 7 DPS. Data from P12 were collected 11 DPS. The *y* axis indicates the values of different biomarkers. Red dots indicate the measure of each patient according to the patient ID plotted in *x* axis.

The precise mechanisms governing the YF pathogenesis and disease outcome are still unknown. It has been proposed that older age, male sex, higher leukocyte and neutrophil counts, higher alanine aminotransferase, aspartate transaminase (AST), bilirubin, and creatinine, prolonged prothrombin time, and higher yellow fever virus RNA plasma viral load were associated with severe YF disease (24). Although the majority of patients with urine detection of YFV RNA presented with altered values for important liver and kidney injuries biomarkers (Fig. 1, Table S1), in accordance with the YF Management book guidelines (25) they all were classified as mild YF disease, with the exception of one patient (ID 12), who was hospitalized in an intensive care unit and died 1 month after hospitalization (Fig. 1, Table S1). The 45 patients without YFV RNA detected in urine were also classified as having mild to severe disease, and they presented similar ranges of biomarkers as observed for patients with YFV RNA in urine (biomarkers presented in Table S2).

**FIG 2:**
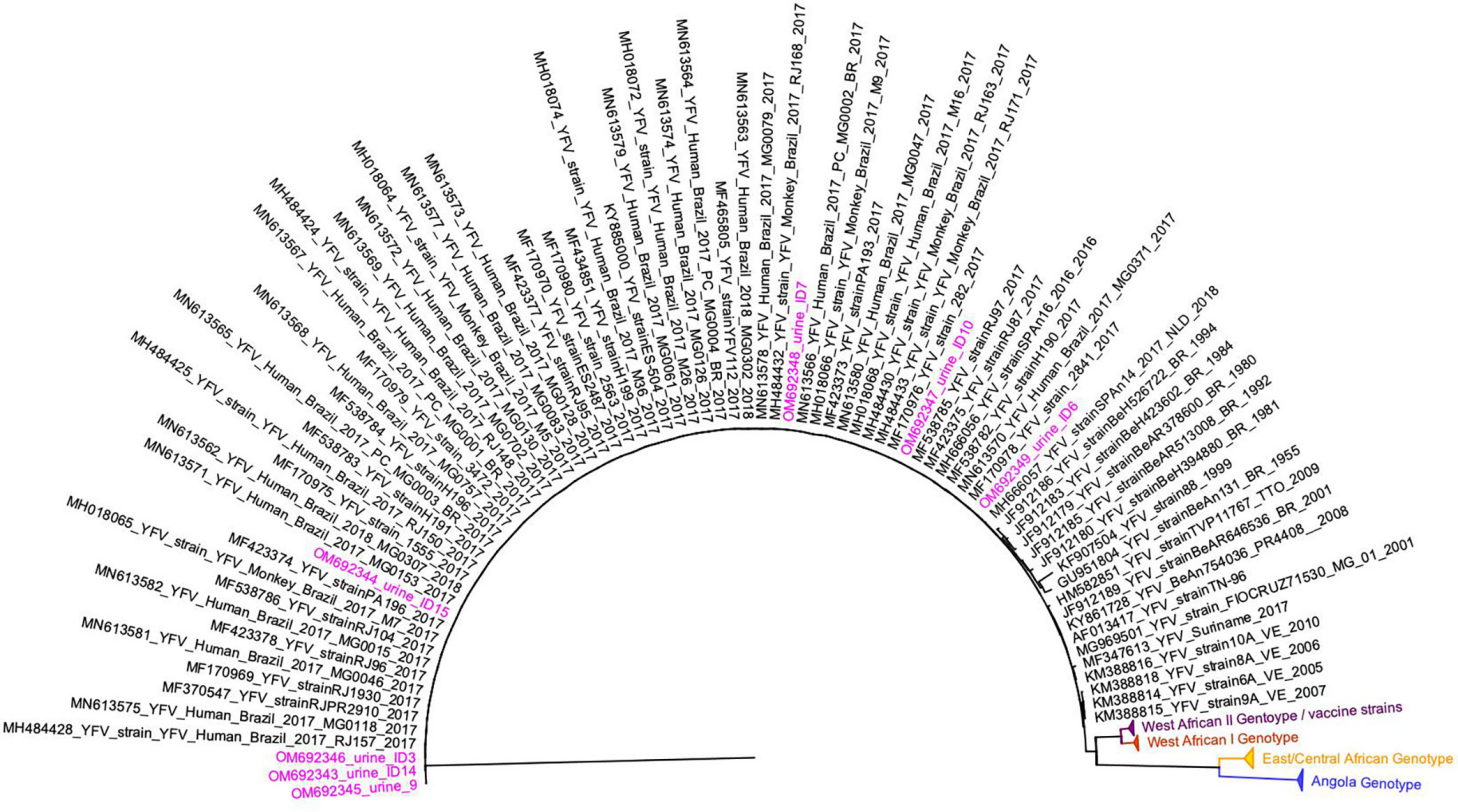
Maximum likelihood tree of yellow fever virus. The maximum clade credibility tree inferred using yellow fever virus (YFV) sequences (213 nt) is shown (corresponding to position 9020 to 9232 compared to the nucleotide sequence of YFV-17D, GenBank accession number: X03700). The bootstrap values (1.000 replicates) are represented by circles drawn in scale in the nodes. Sequences generated in this study are highlighted in pink. The clade containing samples from genotype South American I is represented in black. For clarity purposes, some branches representing different genotypes were collapsed and colored as follows: West African II/vaccine strains (purple), West African I (red), East/Central African (yellow), and Angola (blue). The tree was reconstructed using the nucleotide substitution model kimura 2-parameters with 4-categories gamma distribution. The analysis was performed using MEGAX and the tree visualized and edited in FigTree v1.4.4.

Two urine samples, from the convalescent phase of YF infection (67 and 69 DPS), were collected from patients who received YF vaccine 10 days before symptom onset (ID 14 and ID 15; Table 1) and were genotyped as previously described (20), to investigate possible persistence of the YFV vaccine strain. We were also able to genotype YFV from other three urine samples collected during acute YF infection (GenBank accession number: OM692343 to OM692349) (Table 1 and Fig. 2). Other urine samples were included in this genotype experiment, but sequencing was not successful, likely because of the lower YFV viral load in the used sample. The phylogenetic analyses indicated that all six YFV genotyped samples belonged to the wild-type South American I genotype (Fig. 2), which was associated with the recent outbreaks in Brazil (26–28).

## DISCUSSION

The detection of YFV RNA and South American I genotype in convalescent phase YF samples up to 69 DPS indicates the presence and persistence of YFV RNA in the urine for an extended period of time than previously described. To our knowledge, this is the first report of wild-type YFV RNA presence in the urine of YF patients up to 69 days post-symptom onset. Previous studies had demonstrated the presence of wild-type YFV RNA in the urine up to 32 DPS (16–18).

As recommended by WHO, YF diagnosis must be confirmed by a positive RT-qPCR or with a 4-fold increase in IgG anti-YFV antibody titers between acute and convalescent paired serum samples (12). When retrospective YF outbreaks are under investigation, especially in lower-resourced communities or remote areas, it is often difficult to collect paired serum samples for serologic diagnosis or a serum sample during the short viremic period of YFV. Although international guidelines advocate using YFV molecular tests in sera until 10 days postsymptom onset, the detection of YF RNA in urine during the acute phase of YF indicates the suitability of urine for molecular diagnosis, even when sera are RT-qPCR negative, as demonstrated in this study. Additionally, the limited diagnostic window of YFV RNA detection in sera may be extended with the evaluation of urine samples as demonstrated here and in previous studies (16–18). A few studies had already demonstrated the detection of YFV-17D RNA in urine (29, 30), and with the data presented here, we could suggest that urine can be used as an alternative sample in cases when YFV genotyping is necessary.

Urine collection is a noninvasive, simple, and inexpensive process that could effectively contribute to the investigation of suspected YF cases. The use of urine specimens can extend the window for YFV RNA detection and genotyping with differentiation of wild-type YFV from vaccine virus strains (20). This strategy could be especially useful (i) for retrospective investigation of outbreaks when viremia can no longer be detectable in patients (ii) for investigation of suspected cases of adverse events related to YFV vaccination, (iii) when there is difficulty with phlebotomy as some cases with younger children, or (iv) in conditions lacking proper facilities to process and store biological samples after harvesting. Urine does not need processing after collection (as opposed to the processing of whole blood for obtaining sera), and the inclusion of an RNA stabilizer after collection, if necessary due to collection conditions, can contribute to preservation of urine samples and further enhancement of RNA detection. We understand that one of the limitations of this study is that it was done using samples already collected during the YF outbreak in Brazil. We could not plan an ideal scenario for urine samples collection. Nonetheless, the detection of YFV RNA in urine in 25% of 60 patients tested here supports future prospective cohort studies, providing preliminary data regarding the detection of YFV RNA in urine samples even after 69 days after symptom onset.

Although larger studies are needed, our results from naturally infected YF patients suggest that investigation of YFV RNA in urine can be an effective and supportive additional diagnostic approach, particularly considering the larger detection window compared to serum samples and genotyping suitability. The results of molecular tests using urine as sample for laboratory diagnosis should be carefully interpreted. While a positive result can contribute to YFV diagnosis, a negative (nondetectable) result does not exclude YFV infection.
