# Supplementary material for: Yellow Fever Molecular Diagnosis Using Urine Specimens during Acute and Convalescent Phases of the Disease
Source: J Clin Microbiol. 2022 Aug 2;60(8):e00254-22. doi: 10.1128/jcm.00254-22 (PMC9383191; doi:10.1128/jcm.00254-22)
Supplement: Supplemental file 1 — Tables S1 and S2. Download jcm.00254-22-s0001.pdf, PDF file, 0.1 MB [file jcm.00254-22-s0001.pdf]

| ID | 1 | 2 | 3 | 4 | 5 | 6 | 7 | 8 | 9 | 10 | 11 | 12 | 13 | 14 | 15 |
|---|---|---|---|---|---|---|---|---|---|---|---|---|---|---|---|
| AGE | 31 | 70 | 33 | 64 | 51 | 61 | 48 | 21 | 44 | 44 | 65 | 55 | 52 | 33 | 38 |
| GENDER | male | male | male | male | male | male | male | male | male | male | male | male | male | female | male |
| SYMPTOMS ONSET | 3/3/18 | 1/14/18 | 1/22/18 | 1/28/18 | 2/12/18 | 2/1/18 | 2/26/18 | 2/12/18 | 1/6/18 | 2/9/18 | 1/18/18 | 1/16/18 | 3/15/18 | 1/22/17 | 1/15/17 |
| HOSPITALIZATION DATE | 3/10/18 | 1/17/18 | 1/25/18 | 1/31/18 | 2/15/18 | 2/8/18 | 3/1/18 | 2/15/18 | 1/11/18 | 2/15/18 | 1/24/18 | 1/27/18 | 3/18/18 | 1/27/17 | 3/24/17 |
| DPS | 7 | 3 | 3 | 3 | 3 | 7 | 3 | 3 | 5 | 6 | 6 | 11 | 3 | 5 | 7 |
| CONFUSION | A | A | A | A | A | A | A | A | A | A | A | P | P | A | A |
| HEADACHE | P | P | P | P | P | P | P | A | A | P | P | P | A | P | A |
| MYALGIA | P | P | A | A | A | A | P | P | A | A | P | A | A | P | P |
| FEVER | P | A | A | P | P | P | P | P | P | P | P | P | P | P | P |
| ASTHENIA | P | A | P | P | P | A | P | A | P | P | P | A | A | P | A |
| ABDOMINAL PAIN | A | A | P | A | P | A | A | P | A | A | A | P | A | A | A |
| DIARRHEA | A | A | A | P | A | A | A | A | A | A | A | P | P | A | A |
| VOMIT | P | A | A | P | A | P | A | P | P | A | P | A | P | A | A |
| HEMOGLOBIN (14-18 g/dL) | 15.5 | 14.7 | 14.3 | 16.3 | 14.3 | 15.5 | 13.5 | 12.6 | 16.9 | 14.6 | 14.5 | 10.5 | 12.7 | 13.2 | 16.2 |
| HEMATOCRITE (42 – 50%) | 46.3 | 42.8 | 42.9 | 47.5 | 42.2 | 50 | 41.3 | 39.8 | 51.2 | 46.7 | 44.9 | 39.1 | 46.2 | 43.3 | 47 |
| PLATELETS (150,000-450,000mm$^3$) | 197000 | 130000 | 122000 | 206000 | 98000 | 81000 | 30000 | 151000 | 117000 | 70000 | 170000 | 61000 | 33000 | 109000 | 285000 |
| LEUCOCYTE (4,000 - 11,000 mm$^3$) | 9900 | 2400 | 2500 | 3500 | 2600 | 7200 | 2400 | 1700 | 3970 | 2900 | 5300 | 2600 | 3900 | 2510 | 5400 |
| LYMPHOCYTE (30% - 45%) | 1734 | 1014 | 2530 | 1968 | 1829 | 1152 | 380 | 1080 | 1148 | 1292 | 1610 | 1005 | 1961 | 2420 | 1620 |
| NEUTROPHIL (2,000 - 8,250 mm$^3$) | 6930 | 1272 | 925 | 1722 | 728 | 5328 | 1560 | 784 | 2624 | 1537 | 3337 | 1872 | 2809 | 704 | 3456 |
| EOSINOPHIL (0 – 500 cells/mcL) | 297 | 24 | 50 | 140 | 0 | 144 | 27 | 0 | 88 | 34 | 212 | 52 | 78 | 44 | 162 |

| | | | | | | | | | | | | | | | |
|---|---|---|---|---|---|---|---|---|---|---|---|---|---|---|---|
| CREATININE (0.7 – 1.30 mg/dL) | 1.3 | 1.1 | 1 | 1 | 0.8 | 0.6 | 0.8 | 0.8 | 0.7 | 0.7 | 1 | 1.9 | 0.7 | 0.73 | 0.8 |
| ALBUMIN (3.4 – 5.4 g/dL) | 2 | 4 | 3.8 | 3.8 | 3.8 | 3.1 | 3.9 | 3.9 | 3.6 | 3.6 | 3 | 2.5 | 2 | 3.1 | - |
| AST (10 – 40 U/L) | 1933 | 202 | 37 | 95 | 678 | 268 | 1943 | 645 | 3980 | 379 | 1304 | 7630 | 4350 | 823 | 758 |
| ALT (10 – 40 U/L) | 2300 | 163 | 138 | 100 | 413 | 665 | 1206 | 549 | 3000 | 435 | 1983 | 2390 | 610 | 827 | 374 |
| GGT (9 – 50 U/L) | 318 | 47 | 227 | 93 | 165 | 344 | 42 | 98 | 370 | 229 | 332 | 397 | 689 | 287 | 668 |
| APHOS (30-120 U/L) | 222 | 102 | 115 | 94 | 70 | 74 | 61 | 75 | 144 | 85 | 175 | 187 | 121 | 162 | 131 |
| LACTATE (0.5 – 1 mmol/L) | 1.4 | - | 1.4 | 1.3 | 1.1 | 1.2 | 1.1 | 0.8 | 1.3 | 1.2 | 1.5 | 7.2 | 1.6 | 0.7 | - |
| INR | 1.27 | 1 | 1 | 1 | 1 | 1.01 | 1.2 | 1.09 | 3.9 | 0.7 | 1.63 | 2.01 | 1.14 | 1.01 | - |
| TOTAL BILIRUBIN (0.3 – 1.0 mg/dL) | 11.8 | 0.5 | 0.5 | 0.4 | 0.3 | 2.4 | 0.3 | 1.1 | 3.2 | 0.6 | 10.1 | 9.9 | 4.1 | 2 | 1.6 |
| DIRECT BILIRUBIN (0.1 – 0.3 mg/dL) | 10.3 | 0.4 | 0.3 | 0.3 | 0.3 | 1.6 | 0.3 | 0.4 | 33.5 | 40.5 | 8.1 | 8.6 | 3.4 | 1.5 | 0.9 |
| OUTCOME | DISCAHRGE | DISCAHRGE | DISCAHRGE | DISCAHRGE | DISCAHRGE | DISCAHRGE | DISCAHRGE | DISCAHRGE | DISCAHRGE | DISCAHRGE | DISCAHRGE | DEATH | DISCAHRGE | DISCAHRGE | DISCAHRGE |
| OUTCOME DATE | 3/23/18 | 1/21/18 | 1/30/18 | 2/5/18 | 2/22/18 | 2/15/18 | 3/12/18 | 2/21/18 | 1/16/18 | 2/22/18 | 2/5/18 | 2/26/18 | 4/23/18 | 1/31/17 | 3/30/17 |

Supplementary table 1: Laboratory exams, signs and symptoms at the day of hospitalization of patients attended at Eduardo de Menezes Hospital and confirmed detection of YFV RNA in urine.

DPS: Days post symptoms. A: absecnce. P: presence. AST: aspartate aminotransferase. ALT: alanine aminotransferase. GGT: gamma-glutaryl transferase. APHOS: Alkaline phosphatase. INR: international normalized ratio. Laboratory test reference ranges are following ABIM (2020).

| Biomarker (normal range) | Patients with positive PCR in urine (n=15) | | Patients with negative PCR in urine (n=45) | |
|---|---|---|---|---|
| | Up to 15 DPS (n=13) | From 16 to 94 DPS (n=4) | Up to 15 DPS (n=16) | From 16 to 94 DPS (n=29) |
| AGE (years) | 21 - 69 | 33 - 54 | 21 - 83 | 14 - 80 |
| HEMOGLOBIN (14-18 g/dL) | 12.6 - 16.9 | 10.5 - 16.2 | 6.2 - 16.5 | 7.0 - 18.1 |
| HEMATOCRITE (42 – 50%) | 39.8 - 51.2 | 39.1 - 47.0 | 17.5 - 50.5 | 29.6 - 53.6 |
| PLATELETS (150,000-450,000 $mm^3$) | 30,000 - 206,000 | 33,000 - 285,000 | 9,000 - 224,000 | 9,000 - 398,000 |
| LEUCOCYTE (4,000 - 11,000 $mm^3$) | 1,700 - 9,900 | 2,510 - 5,400 | 1,700 - 9,600 | 1,100 - 13,460 |
| NEUTROPHIL (2,000 - 8,250 $mm^3$) | 728 - 6,930 | 704 - 3,456 | 322 - 6,764 | 612 - 6,300 |
| EOSINOPHIL (0 – 500 cells/mcL) | 0 - 297 | 44 - 162 | 0 - 192 | 0 - 534 |
| CREATININE (0.7 – 1.30 mg/dL) | 0.8 - 1.3 | 0.7 - 1.9 | 0.3 - 4.9 | 1 - 9.9 |
| ALBUMIN (3.4 – 5.4 g/dL) | 2.0 - 4.0 | 2.0 - 3.1 | 1.7 - 4.5 | 2.1 - 4.2 |
| AST (10 – 40 U/L) | 37 - 3,980 | 758 - 7630 | 25 - 5,326 | 37 - 16,220 |
| ALT (10 – 40 U/L) | 100 - 3000 | 374 - 2390 | 25 - 5,326 | 34 - 6,444 |
| GGT (9 – 50 U/L) | 42 - 370 | 287 - 680 | 13 - 24,820 | 73 - 2776 |
| APHOS (30-120 U/L) | 61 - 222 | 121 - 187 | 53 - 413 | 70 - 1070 |
| LACTATE (0.5 – 1 mmol/L) | 0.8 - 1.5 | 0.7 - 7.2 | 1.0 - 6.7 | 1.0 - 3.5 |
| TOTAL BILIRUBIN (0.3 – 1.0 mg/dL) | 0.3 - 11.8 | 1.6 - 9.9 | 0.4 - 26.6 | 0.5 - 9.8 |
| DIRECT BILIRUBIN (0.1 – 0.3 mg/dL) | 0.3 - 40.5 | 0.9 - 8.6 | 0.2 - 22.6 | 0.1 - 8.9 |
| INR | 0.7 - 1.63 | 1.01 - 2.01 | 1.0 - 5.11 | 1.0 - 5.0 |

Supplementary table 2: Laboratory exams range at the day of hospitalization of patients attended at Eduardo de Menezes Hospital with tested urine sample. Values from patients with positive urine sample were highlighted in light grey.

DPS: Days post symptoms. AST: aspartate aminotransferase. ALT: alanine aminotransferase. GGT: gamma-glutaryl transferase. APHOS: Alkaline phosphatase. INR: international normalized ratio. Laboratory test reference ranges are following ABIM (2020).
